# Annealing Behaviour of Pt and PtNi Nanowires for Proton Exchange Membrane Fuel Cells

**DOI:** 10.3390/ma11081473

**Published:** 2018-08-19

**Authors:** Peter Mardle, Shangfeng Du

**Affiliations:** School of Chemical Engineering, University of Birmingham, Edgbaston, Birmingham B15 2TT, UK; PJM556@student.bham.ac.uk

**Keywords:** proton exchange membrane fuel cell (PEMFC), electrocatalyst, PtNi, nanowire, annealing

## Abstract

PtNi alloy and hybrid structures have shown impressive catalytic activities toward the cathodic oxygen reduction reaction (ORR) in proton exchange membrane fuel cells (PEMFCs). However, such promise does not often translate into improved electrode performances in PEMFC devices. In this contribution, a Ni impregnation and subsequent annealing method, translatable to vertically aligned nanowire gas diffusion electrodes (GDEs), is shown in thin-film rotating disk electrode measurements (TFRDE) to enhance the ORR mass activity of Pt nanowires (NWs) supported on carbon (Pt NWs/C) by around 1.78 times. Physical characterisation results indicate that this improvement can be attributed to a combination of Ni alloying of the nanowires with retention of the morphology, while demonstrating that Ni can also help improve the thermal stability of Pt NWs. These catalysts are then tested in single PEMFCs. Lower power performances are achieved for PtNi NWs/C than Pt NWs/C. A further investigation confirms the different surface behaviour between Pt NWs and PtNi NWs when in contact with electrolyte ionomer in the electrodes in PEMFC operation. Indications are that this interaction exacerbates reactant mass transport limitations not seen with TFRDE measurements.

## 1. Introduction

In order for proton exchange membrane fuel cells (PEMFC) to become viable for full scale commercialisation, the catalyst activities and utilisation ratio of the precious metal catalysts, i.e., Pt/C in catalyst electrodes needs to increase to improve the fuel cell power performance and reduce the system cost. To address this challenge, a lot of work has been conducted on the development of electrocatalysts toward the cathodic oxygen reduction reaction (ORR) for PEMFC applications over the past decades [1], and mass activities of above 30 times over the commercial Pt/C catalyst have been reported [2,3,4]. However, due to the complex environment and unclear behaviour in operating electrodes, many novel electrocatalysts show poor performance in actual PEMFC operation despite much improved intrinsic catalytic activities demonstrated by the liquid half-cell measurement. A drive for PEMFCs at high current density operation [5,6] means that this end application needs to be more considered during the catalyst development.

One-dimensional (1D) Pt-based alloy and hybrid structures have been demonstrated showing remarkable catalytic activities for a variety of fuel cell reactions [7,8,9]. Concerning the ORR, alloying Pt with a non-precious metal such as Ni or Co, etc. can provide inherent catalytic benefits resulting from lattice strain and electronic effects. They reduce the Pt–O bond strength and thus shift the catalytic activity towards the theoretical maximum [10,11]. Additionally, Pt nanowires (NWs) have shown much higher specific activities toward the ORR in comparison to their 0D counterparts, particularly because of the preferential exposure of crystal facets with single crystal nanowires [12,13]. Hence, a combination of the 1D morphology with alloy/hybrid structures has attracted much attention. Fundamental materials research has been conducted by Bu et al. who developed a variety of ultra-thin, long and crystalline PtM (M = Ni, Co, Fe, and Rh) NWs by a one-pot synthetic route, which demonstrated extraordinarily high catalytic activities for the ORR [3]. Utilising the Pt NW structure formed by the formic acid reduction method on carbon nanotubes, Elvington et al. deposited Ni onto the surface of Pt NWs by use of hydrazine monohydrate and sodium hydroxide [14]. The Ni was subsequently annealed into the Pt by means of thermal treatment in a reductive environment to provide a highly active catalyst for the ORR.

Alia et al. also produced ultra-long PtNi NWs by means of galvanic displacement of commercial Ni NWs with Pt [7]. The as-synthesised PtNi NWs showed 3-fold improvements in mass activity for the ORR over Pt NWs, aided by the retention of high electrochemically active surface areas (ECSA). Post treatment, such as thermal annealing [15] and acid treatment [16], have also been investigated to tailor these materials for practical use in PEMFCs. However, a large disparity is still observed between the intrinsic catalytic activity of the PtNi NWs and their performance in electrodes in fuel cells [17]. Therefore, to progress the development of high performance PEMFC devices from 1D Pt-based structures, their behaviour in practical electrodes need to be further understood on top of the improved inherent ORR catalytic activity.

Work previously conducted in our group has demonstrated that aligned Pt NWs can be grown directly on the carbon paper gas diffusion layer (GDL) by a simple and scalable formic acid reduction approach [18,19]. With the refinement of the growth temperature and by introducing nano-seeds on the GDL surface during the synthesis, Pt NW gas diffusion electrodes (GDEs) with better performances were achieved in comparison to conventional ones with the commercial Pt/C catalysts [19,20]. If analogous electrodes with similarly aligned PtNi NWs can be achieved, it can then potentially further enhance the fuel cell performance in applications.

In this work, a variety of PtNi NWs supported on carbon black (Vulcan XC-72R) are produced to understand the catalyst behaviour in fuel cell electrodes as a feasibility study. Pt NWs are grown on the carbon black particles using the formic acid approach at room temperature [21]. Ni is then deposited onto the surface of the Pt NWs using sodium borohydride in a simple wet chemical approach. Both PtNi and Pt NWs supported on carbon are then thermally annealed under a flow of H_2_. The effect of both annealing temperature and time on the morphology and structure of Pt and PtNi NWs are discussed. The relation between the intrinsic activities of the 1D NWs determined by thin-film rotating disk electrode (TFRDE) measurement and their power performance in operating PEMFCs is also explored.

## 2. Experimental

### 2.1. Materials

H_2_PtCl_6_·6H_2_O, HCOOH (≥95%), NiCl_2_·6H_2_O, and NaBH_4_ were purchased from Sigma-Aldrich (Gillingham, Dorset, UK). Vulcan XC-72R carbon black was purchased from the Fuel Cell Store (College Staion, TX, USA). Ethanol was purchased from Fisher Scientific (Loughborough, Leicestershire, UK). 10 M NaOH from Fluka Biochemika was used and diluted to 0.1 M in-house. 10% Nafion^®^ solution (D1021) was obtained from Ion Power Inc. (Munich, Bavaria, Germany). All H_2_O was deionised to 18 MΩ cm using a Millipore water system. For the liquid half cell measurement and fuel cell test, a benchmark commercial Pt/C catalyst (45.9 wt. % Pt, TEC10E50E, Tanaka KikinzokuKogyo K. K. (TKK)) was used.

### 2.2. Preparation of Pt NWs/C

To prepare 60 wt. % Pt NWs/C catalysts, 9.0 mL of 8 wt. % H_2_PtCl_6_ aqueous solution was added to a dispersion of 240 mg Vulcan XC-72R in 750 mL H_2_O. 48 mL of HCOOH was then added dropwise within 25 min under sonication. The reaction mixture was then left to react for 72 h before washing with H_2_O and then ethanol by centrifugation. The Pt NWs/C were then dried overnight in an oven at 60 °C.

### 2.3. Preparation of PtNi NWs/C

To impregnate Pt NWs/C with Ni, 300 mg of the as-prepared Pt NWs/C was dispersed in a solution of 73 mg NiCl_2_·6H_2_O in 180 mL 0.1 M NaOH to achieve a theoretical Pt:Ni atomic ratio 3:1. 80 mg of NaBH_4_ was then dissolved in 40 mL of 0.1 M NaOH before adding dropwise to the Pt NW/C and NiCl_2_ dispersion under vigorous stirring. After 1 h, the PtNi NWs/C were washed and dried as above with the Pt NWs/C samples.

### 2.4. Annealing of Pt NWs/C and PtNi NWs/C

Pt NWs/C and PtNi NWs/C were thermally annealed at 150, 250, and 350 °C for 24 h under a 50 mL min^−1^ flow of 4% H_2_/Ar in a tube furnace (Vecstar Ltd. Chesterfield, Derbyshire, UK) with a heating and cooling rate of 2 °C min^−1^. A PtNi NWs/C sample was also annealed at 150 °C for 72 h to study the influence of annealing duration. The annealing temperature used is stated in brackets throughout the report.

### 2.5. Physical Characterisation

Transmission electron microscopy (TEM) and energy dispersive X-ray spectroscopy (EDX) analyses of the Pt NWs/C and PtNi NWs/C before and after annealing were conducted with a Jeol2100 FEG-TEM (JEOL Ltd. Welwyn Garden City, Hertfordshire, UK) at an accelerating voltage of 200 kV to assess the morphology and chemical composition of the NANOWIREs. In order to assess the crystallinity and Ni alloying degree in the PtNi NWs/C samples, powder X-ray diffraction (XRD) was recorded using a Bruker D8 Auto-sampler (Bruker Corp. Billerica, MA, USA), utilising a Cu Kα X-Ray source (λ = 0.15406 nm). The XRD was ran between 2θ values of 20–90° with a step size of 0.02° and dwell time of 0.47 s. X-ray photoelectron spectroscopy (XPS) was conducted with an XPS spectrometer (K-Alpha, Thermo Scientific, Loughborough, Leicestershire, UK) using a micro-fused monochromatic Al Kα source (1486.6 eV). Thermo-gravimetric analysis (TGA) was conducted with a NETZSCH TG209F1 (NETZSCH GmbH. Selb, Bavaria, Germany) in the temperature range 20–700 °C at a heating rate of 10 °C min^−1^ under a 40 mL min^−1^ air flow.

### 2.6. Thin-Film Rotating Disk Electrode Measurement (TFRDE)

TFRDE measurements were performed to evaluate the catalyst activities using a standard 3 electrode rotating disk electrode (RDE) setup with an Autolab PGSTAT302N potentiostat (Metrohm Ltd. Runcorn, Cheshire, UK). 5 mg_Catalyst_ mL^−1^ catalyst ink with volume ratios H_2_O:IPA:Nafion^®^ 0.79:0.2:0.01 was homogenised using both ultra-sonic cleaner and horn. A Sonics Vibra-CellTM VCX130 sonication horn was used at 20% power for 10 min sonic time in a 10 s on/10 s off pulse sequence. Five μL of the fresh ink was pipetted onto a 0.196 cm^2^ Pine Instruments (Pine Research Instrumentation Inc. Durham, County Durham, UK) glassy carbon electrode (GCE) which was pre-polished using alumina slurries. The electrode was then left for drying under a rotation speed of 600 rpm in ambient conditions thus ensuring a homogeneous thin film paramount for an accurate comparison of catalyst samples [22,23]. An in-house prepared reference hydrogen electrode (RHE) and Pt gauze were used as the reference and counter electrodes respectively. 0.1 M HClO_4(aq)_ was used as the electrolyte and was maintained at 25 °C by a water bath. After the electrolyte was saturated with N_2_, 50 cyclic voltammograms (CVs) were run in the potential range 0.05–1.2 V at a scan rate of 100 mV s^−1^ to electrochemically clean the surface followed by 3 scans at 20 mV s^−1^ (staircase, 0.00244 V). The hydrogen desorption region (H_des_) of the final scan was used to calculate the electrochemical effective surface area (ECSA) although it is noted that use of a staircase scan could underestimate the true value due to the fast hydrogen adsorption/desorption process [24]. Kinetic currents were then obtained by means of a Koutecky-Levich analysis from background and internal resistance corrected linear sweep voltammograms (0.05–1.2 V vs. RHE, 20 mV s^−1^) in O_2_ saturated electrolyte at rotation rates of 400, 800, 1200, 1600, and 2000 rpm. Specific and mass activities were calculated by normalising the kinetic current with respect to electrochemically active Pt surface area and Pt mass, respectively. The mass was calculated assuming 60 wt. % for Pt NWs/C samples and 56.6 wt. % for PtNi NWs/C samples. The internal resistances were determined from electrochemical impedance spectroscopy (EIS) at the intersection point with the real axis.

### 2.7. Membrane Electrode Assemble (MEA) Preparation and Test

Typically, catalyst electrodes were made by firstly preparing a catalyst ink of 14 mg of catalyst (0.4 mg_Pt_ cm^−2^ in final electrodes, ca. 24% lost in painting) in 0.5 mL IPA and 90.5 μL Nafion^®^ solution, by use of sonication. The ink was painted onto a 4 × 4 cm^2^ piece of Sigracet 35BC GDL and dried in air. Membrane electrode assemble were then fabricated by hot pressing the as-prepared electrodes with a 6 × 6 cm^2^ Nafion 212 membrane and a commercial Johnson Matthey anode (0.4 mg_Pt_ cm^−2^) on which a thin layer of Nafion^®^ ionomer had been coated in advance. Hot pressing was carried out at a pressure of 4.9 MPa at 135 °C for 2 min.

The fabricated MEAs were tested at 80 °C in a PaxiTech-BioLogic FCT-50S PEMFC test stand (PaxiTech SAS. Grenoble, Auvergne-Rhône-Alpes, France). Polytetrafluoroethylene (PTFE) gaskets with a thickness of 254 μm were used at both the anode and cathode. The break-in procedure and polarisation curve acquisition followed the harmonised EU protocol [25]. For the polarisation curves and EIS, the air/H_2_ relative humidity (RH), stoichiometric coefficient and absolute pressure was 30%/50%, 1.5/1.3, and 2.3/2.5 bar, respectively. Galvano EIS was conducted at 0.48 A with amplitude 72 mA in the frequency range 10–0.1 Hz. Potentio EIS was conducted at 0.65 V and 0.5 V with amplitude 10 mV in the same frequency range as with the galvano EIS measurement.

## 3. Results and Discussion

### 3.1. Physical Characterisation

**Figure 1:**
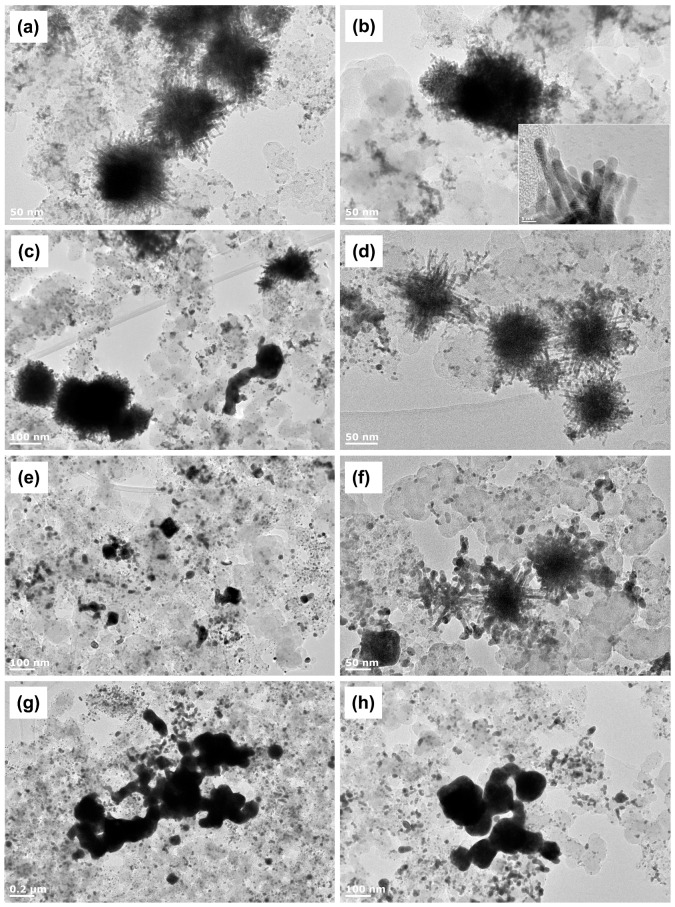
TEM images of (**a**) Pt nanowires (NWs)/C; (**b**) PtNi NWs/C; (**c**) Pt NWs/C (150 °C); (**d**) PtNi NWs/C (150 °C); (**e**) Pt NWs/C (250 °C); (**f**) PtNi NWs/C (250 °C); (**g**) Pt NWs/C (350 °C); and (**h**) PtNi NWs/C (350 °C). Inset 1b show the HR-TEM images of the PtNi NWs.

Figure 1 shows TEM images of Pt NWs/C and PtNi NWs/C samples before and after annealing at various temperatures. Figure 1a shows that while a lot of singular Pt NWs grow on the carbon surface, the majority of the nanowires are formed into large agglomerates with an average size of ca. 120 nm. Based on the similarities of these agglomerates with previous investigations of Pt NWs grown using formic acid, it seems that the size and shape of the Pt NW agglomerates depends primarily on the underlying seed formed on the support [21,26], and the nanowire growth rate [27]. Control of the growth temperature [19], the separate introduction of seed particles [26], and a variety of other factors such as precursor selection [28] can give better control over this agglomeration. However, the optimisation procedure of the growing process in large quantities is beyond the research topic in this work, considering the principle investigation here is the annealing behaviour. Of more importance is the fact that all samples are derived from the exact same initial batch of Pt NWs/C, thereby all differences found from the TFRDE and MEA tests are solely due to the inclusion of Ni and annealing temperature used, and not differences in initial Pt NW structure and distribution.

The individual Pt NWs (Figure 1a) as part of the agglomerates have an average diameter of 3.40 nm. Based on a measured inter-lattice spacing of 0.22 nm, evidence suggests NW growth in the <111> direction, leading to the preferential exposure of highly active crystal facets. Upon the impregnation of Ni, the wire diameter increased to 3.59 nm and the inter-lattice spacing remained at 0.22 nm. EDX element mapping were also run and the results for one PtNi NWs/C sample is shown in Figure S1 where the distribution of Ni throughout the agglomerate is clear however resolution was not sufficient to show the distribution of Ni on an individual nanowire.

The TEM images of the Pt NWs/C and PtNi NWs/C samples under various annealing temperatures are also shown in Figure 1. Under a mild annealing temperature of 150 °C, the nanowire diameters of the Pt NWs/C and PtNi NWs/C are found to be 5.06 nm and 3.56 nm, respectively. Shown in Figure 1c is also an instance where sintering of Pt NWs/C had occurred. Figure 1d indicates that at this annealing temperature, the morphology of PtNi NWs/C can be completely retained, thus evidencing the presence of Ni on the Pt nanowires. At a higher annealing temperature of 250 °C, the vast majority of Pt NWs and PtNi NWs have sintered, and an average diameter of 8.58 nm are found for the PtNi NWs/C. At 350 °C, severe coarsening happens and the large nanowire agglomerates sinter into large particles. This result demonstrates both the mobility of the Pt on the carbon surface at temperature [29] and critically that the ultra-high annealing temperatures of up to 900 °C that can be used for supported nanoparticles [30,31] cannot be used if the ultra-thin nanowire morphology needs to be retained. To further understand the influence of annealing duration, annealing was conducted for PtNi NWs/C for 72 h. The average nanowire diameter after annealing is ca. 3.53 nm (Figure S2) indicating that a longer annealing time does not result in severe sintering.

**Figure 2:**
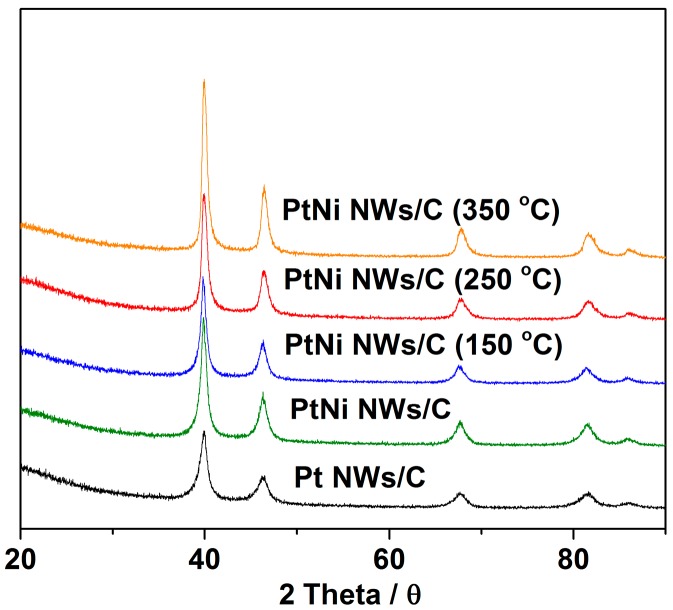
XRD patterns of Pt NWs/C and PtNi NWs/C.

XRD analysis was conducted to the PtNi NWs/C samples in order to compare the degree of PtNi alloying at each annealing temperature (Figure 2). The XRD patterns of Pt NWs/C, PtNi NWs/C, and PtNi NWs/C (150 °C) all have similar peak positions to the pure crystalline Pt with calculated lattice spacings of 3.913, 3.914, and 3.917 Å respectively. It is only at the higher annealing temperatures of 250 and 350 °C, with respective lattice constants of 3.908 and 3.906 Å that a notable shift in lattice constant is observed. However, this shift is much smaller in comparison to other PtNi studies [32,33] demonstrating that the alloying here only occurs below the Pt surface, not affecting the bulk nanowire composition [14]. No characteristic Ni peaks are found in the XRD patterns, indicating that the Ni successfully incorporates into the Pt lattice structure in the PtNi NWs/C samples. From the XRD patterns an increase in peak intensity is also observed corresponding to an increase in particle size, consistent with TEM analysis results above [33].

**Figure 3:**
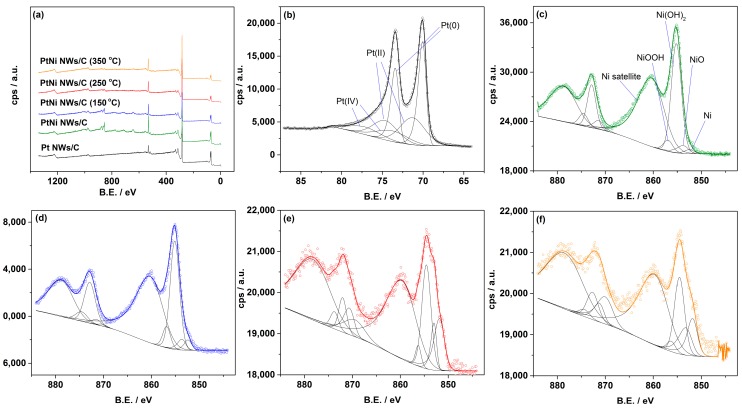
(**a**) XPS Survey scan of Pt NWs/C, PtNi NWs/C, and PtNi NWs/C annealed at various temperatures. XPS patterns of (**b**) Pt 4f scan for Pt NWs/C; (**c**) Ni 3p scan for PtNi NWs/C; (**d**) Ni 3p scan for PtNi NWs/C (150 °C); (**e**) Ni 3p scan for PtNi NWs/C (250 °C); and (**f**) Ni 3p scan for PtNi NWs/C (350 °C).

XPS analysis of the Pt NWs/C and Pt NWs/C samples was also performed to ascertain the nature of the Pt and Ni in each sample (Figure 3 and Figure S3). For all samples, the best fit for Pt 4f peaks is found with the inclusion of both PtO and PtO_2_ (Table S1). For the high resolution Ni regions, Ni(OH)_2_ is found to be the largest contributor to the non-satellite peaks. However, the Ni(OH)_2_ content decreases and the Ni content increases with the annealing temperature (Table S2). This supports the XRD analysis results with the conclusion that higher degrees of alloying are obtained at the higher annealing temperatures, where Pt surface segregation is more prominent and reduction of the Ni hydroxides by the H_2_ gas leads to sub-surface Ni [34]. The sintering of the nanowires is also a cause in positioning the Ni within the large crystalline agglomerates of Figure 1h.

Evidence of a larger proportion of surface Ni hydroxides is also found for the PtNi NWs/C and PtNi NWs/C (150 °C) using thermogravimetric analysis (TGA). A drop in residual mass at around 200–300 °C in the TGA (Figure S4) is ascribed to the decomposition of surface hydroxide species [35]. However, no such drop is observable for the samples annealed at 250 °C and 350 °C, coinciding with the much reduced hydroxide content found in the XPS analysis. The residual mass of 61% for Pt NWs/C and 55% for PtNi NWs/C annealed at 350 °C are very close to the assumed Pt loadings of 60 wt. % for the Pt NWs/C samples and 56.6 wt. % for the PtNi NWs/C samples, respectively.

### 3.2. Ex-Situ ORR Catalytic Activities

TFRDE tests have been extensively used to evaluate the inherent catalytic activity of a material towards ORR [36], and in this work they were used to evaluate the effect of the annealing temperature on the ECSA and catalytic performance of Ni impregnated Pt NW catalysts. A full Koutecky–Levich analysis (Figure S5) was used in this work over the conventionally used mass transport correction due to the fact that the current at 0.9 V vs. RHE was not always less than i_d_/2, where i_d_ is the diffusion limited current [22,37].

**Figure 4:**
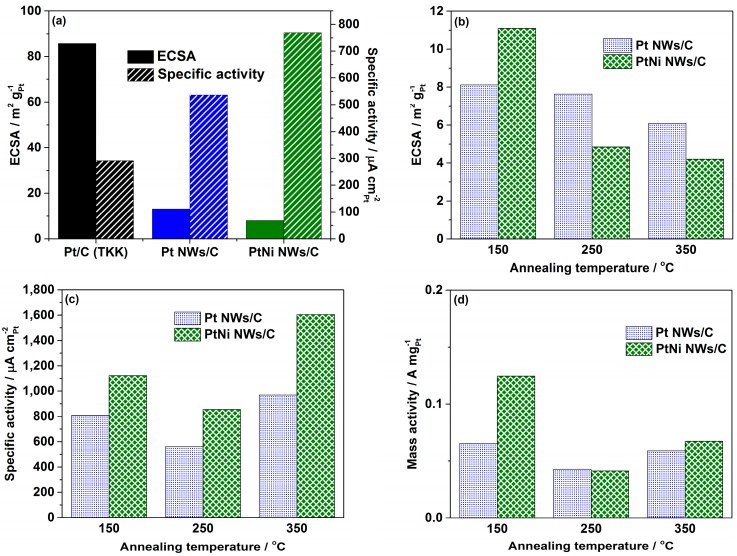
(**a**) Electrochemically active surface areas (ECSA), specific, and mass activity of Pt/C (TKK), Pt NWs/C, and PtNi NWs/C; (**b**) ECSA; (**c**) Specific activity and (**d**) Mass activity of annealed samples of Pt NWs/C, and PtNi NWs/C determined from the thin-film rotating disk electrode measurements (TFRDE) measurement.

Figure 4a shows a comparison of the obtained ECSAs and specific activities for the commercial Pt/C (TKK) catalyst and the Pt NWs/C before and after Ni impregnation. For the commercial catalyst an ECSA and specific activity of 85.7 m^2^ g_Pt_^−1^ and 292 μA cm_Pt_^−2^ are obtained meeting the expected standard [22]. However for the Pt NWs/C the heavy agglomeration results in a small ECSA of 13.1 m^2^ g_Pt_^−1^, and the surface Ni impregnation reduces this to 8.0 m^2^ g_Pt_^−1^. The TGA and XPS analysis has shown a high level of Ni hydroxides suggesting that without the annealing process, the Ni resides on the surface of the Pt nanowires, partially blocking otherwise catalytically active sites on the surface. For the Pt NWs/C and PtNi NWs/C catalysts, specific activities of 536 μA cm_Pt_^−2^ and 769 μA cm_Pt_^−2^ are obtained respectively. Based on previous studies, such increases in the specific activity can be attributed to the inherent benefits of 1D morphology while the Ni contributes to a further boost in activity through electronic and structural effects [10,38].

Of interest is the effect that the annealing temperature has on the catalytic performance of the nanowire catalysts. As a result of the sintering present at a temperature as low as 150 °C, the ECSAs of the Pt NWs/C samples drop from 13.1 m^2^ g_Pt_^−1^ before annealing to 8.1, 7.6, and 6.1 m^2^ g_Pt_^−1^ after annealing at 150, 250, and 350 °C respectively. However, while PtNi NWs/C had an ECSA of 8.0 m^2^ g_Pt_^−1^, after annealing at 150 °C, the ECSA increases to 11.1 m^2^ g_Pt_^−1^. It appears that on annealing, some of the impregnated Ni segregates below the Pt surface, unblocking some Pt active sites while retaining nanowire morphology. However, despite improved resistance to agglomeration in comparison to Pt NWs/C as suggested by the TEM analysis, the ECSA of the PtNi NWs/C annealed at 250 and 350 °C decrease to be 4.8 and 4.2 m^2^ g_Pt_^−1^ respectively. A contributing factor can be an underestimation of the ECSA due to modified electronic properties of the Pt surface [39]. An ECSA of 9.3 m^2^ g_Pt_^−1^ is obtained for PtNi NWs/C annealed at 150 °C for 72 h., supporting the observation that while the nanowire morphology is stable at 150 °C for extended periods of time (Figure S2), the degree of nanowire agglomeration still increases.

Upon annealing at 150 °C, the specific activity of Pt NWs/C and PtNi NWs/C increase to respective values of 808 and 1123 μA cm_Pt_^−2^ (Figure 4c). For the Pt NWs/C the sintering of defect sites is attributable to this increase in observable activity. Resulting from the loss of preferential exposure of highly active facets, after annealing at 250 °C the specific activity for Pt NWs/C is found to be 561 μA cm_Pt_^−2^, less than the untreated Pt NWs/C. An increase in specific activity to 968 μA cm_Pt_^−2^ at the highest studied annealing temperature of 350 °C however can be attributed to the increase in particle size and a reduced proportion of defect sites to crystal faces [40].

A similar relation of annealing temperature to specific activity is observed for the PtNi NWs/C samples where specific activities of 1123, 855, and 1606 μA cm_Pt_^−2^ are observed after annealing at 150, 250, and 350 °C respectively. Longer annealing times at 150 °C negatively affected the activity of the PtNi NWs/C where a specific activity of 889 μA cm_Pt_^−2^ is obtained. With higher activities in comparison to the Pt NWs/C analogues, these tests have shown that the impregnation with annealing process can be used to increase the inherent specific catalytic activities of Pt NW catalysts toward the ORR. Of particular importance for practical applications is however the mass activity. Although the mass activity of all prepared samples falls short of the commercial Pt/C (TKK) catalyst, this is a direct consequence of the lower ECSAs and absolute surface area of Pt, itself resulting from the aforementioned commitment to use of the same PtNW/C batch for accurate interpretation of the effect of thermal annealing.

At higher annealing temperatures, the mass activity of the synthesised catalysts is not much improved for the presence of Ni due to the lower ECSAs. However, the retention of nanowire morphology for PtNi NWs/C (150 °C), and an impressive specific activity deriving from a combination of the Ni alloying and the removal of defect sites results in a 1.78-fold mass activity improvement over the initial Pt NWs/C catalysts. Therefore, PtNi NWs/C annealed for 24 h at 150 °C is selected as a candidate material for the fabrication of the high performance PtNi NW electrodes [19,41].

### 3.3. MEA Testing

**Figure 5:**
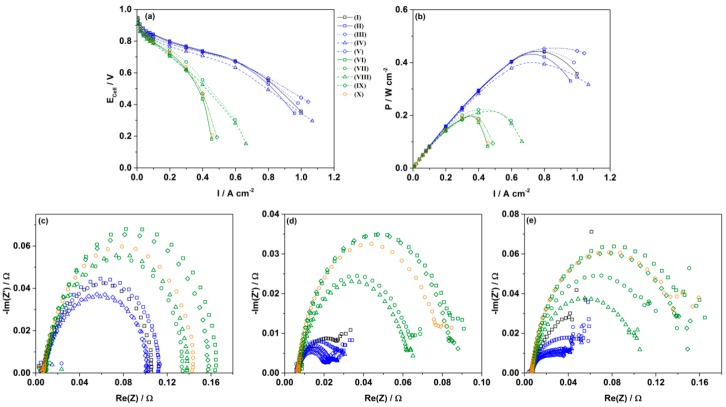
(**a**) Polarisation curves; (**b**) Power density curves and (**c**–**e**) EIS conducted at 0.48 A, 0.65 V, and 0.5 V respectively on electrodes of (I) Pt/C (TKK); (II) Pt NWs/C (III) Pt NWs/C (150 °C); (IV) Pt NWs/C (250 °C); (V) PtNWs/C (350 °C); (VI) PtNi NWs/C; (VII) PtNI NWs/C (150 °C); (VIII) PtNi NWs/C (250 °C); (IX) PtNi NWs/C (350 °C); and (X) PtNi NWs/C (150 °C, 72 h).

In order to better understand the annealing effect, and to discern whether the activity enhancements demonstrated in the TFRDE measurements can be translated into higher electrode power outputs, the as-prepared catalysts were made into 16 cm^2^ GDEs and tested in single PEMFCs (Figure 5). In single cells, the open circuit potential (OCP) of all samples are comparable to each other and notably the activity of the catalysts at 0.9 V are not enhanced too much by the inclusion of Ni in the samples. One of the main challenges for PEMFC development is in realising the promising catalytic activities of novel PtNi catalysts in a working PEMFC. For this purpose, a much deeper understanding of the characteristics of PtNi alloys in MEAs is imperative.

Of more concern than the similar power performance at high potential for Pt and PtNi NWs/C is the drop in performance at higher current densities for the PtNi NWs/C samples. At higher current densities, mass transport limitations become prominent, leading to a sudden drop in cell potential and power output. From the Pt NWs/C samples it appears that the severe coarsening of the nanowires does not change the electrode performance as much as might be predicted. Considering the agglomeration of the nanowires, the Nafion^®^ ionomer in catalyst electrodes is unable to access the entire surface of each nanowire [42]. This implies that in a MEA, each nanowire agglomerate would show similar mass transport characteristics as a single large particle hence giving similar electrode power performances before and after the annealing process. Such results reiterate the importance of dispersion of 1D structures to maximise the triple-phase boundary and to reduce mass transport resistances in electrodes.

To illustrate this further, the sample of PtNi NWs/C annealed at 150 °C for 72 h shows a higher mass transport resistance compared to the PtNi NWs/C annealed for 24 h despite a similar charge transfer resistance (Figure 5c,e). This result can be attributed to the increased aggregation despite similar nanowire diameters found for this sample. Therefore while nanowire morphology is important for improving mass transport limitations in PEMFCs, of greater importance is the distribution of those nanowires.

A more stark difference in performance is however observed when comparing those samples containing Ni with the analogous samples of pure Pt. With Ni impregnation much lower power outputs are obtained in comparison to the Pt NWs/C samples. EIS (Figure 5c–e) confirms that both charge transfer and mass transport resistances for the PtNi NWs/C samples are higher in comparison to the Pt NWs/C samples despite improved catalytic activities observed in liquid half-cell measurement and the higher resistance to coarsening effects in annealing process. While in the TFRDE measurement the ECSA and mass activity can be used to ascertain the Pt surface to bulk ratio and surface activity, in a fuel cell device the catalytic activity more specifically represents the number of surface sites with access to protons to form triple phase boundary (TPB) in electrodes [6,43]. This depends on the morphology and catalytic activities of the catalyst, the distribution of Nafion^®^ ionomer and also on the surface properties of the nanowires. PtNi catalyst surface usually shows a different hydrophilic feature as compared with the hydrophobic surface of pure Pt catalyst [44], and this can potentially lead to a much different contact with Nafion^®^ ionomer in the catalyst layer and the distribution of produced water in fuel cell operation, thus altering the TPB and power performance. Furthermore, cation contaminants are known to exchange with the sulfonic groups of Nafion^®^, altering not just the ionic conductivity, but also the oxygen diffusion coefficient in the ionomer which can exacerbate mass transport limitations in the catalyst layer [45].

It cannot be ruled that that some of the disparity between the activities with the TFRDE and the MEAs could also be due to different stabilities of the Ni from dissolution in each environment, where thicker Pt surface layers are required to protect the sub surface Ni in an MEA environment [6]. While the structural changes of the wires after leaching in each environment and the introduction of a de-alloying step is the focus of an ongoing study, this report serves to provide insights to the annealing behaviour of nanosized 1D Pt and PtNi catalysts at various temperatures and the immediate consequences on catalyst and single cell performance, with the addition of highlighting the challenges facing the adoption of similar novel catalyst materials in PEMFC electrodes.

## 4. Conclusions

A method of fabricating PtNi nanowires in a manner that can be adopted for any pre-existing Pt structure has been demonstrated in this work through an impregnation and subsequent annealing approach. The annealing behaviour of these nanosized 1D catalysts has been evaluated where it is shown that the Pt NWs/C sinters at an annealing temperature as low as 150 °C. Therefore, while the surface cover of Ni onto the ultra-thin Pt NWs increases their thermal stability, only very low annealing temperatures can be employed on nanosized 1D Pt structures if the original morphology is to be retained. Physical characterisation in conjunction with liquid half-cell RDE measurements show that while higher annealing temperatures are required to increase the Pt lattice strain through an increase in Pt surface segregation in Ni impregnated samples, the retention of nanowire morphology for PtNi NWs/C at 150 °C provides a 1.78-fold mass activity improvement to the synthesised Pt NWs/C catalyst. The big differences in ECSA and mass activities detected in liquid half-cell measurement with the annealing temperature and duration results in very limited changes to the power performance of both Pt NWs/C and PtNi NWs/C electrodes in single PEMFC tests. Furthermore, worse performance is found for those samples with Ni resulting from the larger charge transfer and mass transport resistances. Indications are that the contact between the catalysts and electrolyte ionomer, and the surface Ni hydroxide species play a significant role in this poor performance although the precise mechanisms need to be investigated further. Acid leaching/dealloying is proposed to help alleviate the negative impact of surface Ni hydroxide species and will be crucial in realising the potential of novel PtNi catalyst structures to produce real high performance PEMFC devices.

## Supplementary Materials

The following are available online at http://www.mdpi.com/1996-1944/11/8/1473/s1, Figure S1: STEM mode image and EDX element map of PtNi NWs/C annealed at 350 °C. The EDX map is of Pt (green) and Ni (red), Figure S2: TEM of PtNi NWs/C (150 °C, 72 h), Figure S3: XPS patterns of the Pt 4f regions of (a) PtNi NWs/C, (b) PtNi NWs/C (150 °C), (c) PtNi NWs/C (250 °C), (d) PtNi NWs/C (350 °C), (e) PtNi NWs/C (150 °C, 72 h) and (f) the Ni 3p region of PtNi NWs/C (150 °C, 72 h), Figure S4: FFT 5 point smoothed TGA of (I) Pt NWs/C, (II) PtNi NWs/C, (III) PtNi NWs/C (150 °C), (IV) PtNi NWs/C (250 °C) and (V) PtNi NWs/C (350 °C), Figure S5: (a) CVs of 25 μg catalyst on a 0.196 cm^2^ GCE in N^2^ saturated 0.1 M HClO_4(aq)_ electrolyte in the potential range 0.05–1.2 V. (b) LSVs at 1600 rpm in O^2^ saturated electrolyte from 0.05–1.2 V vs. RHE with a sweep rate of 20 mV s-1. (c) Koutecky-Levich plots. The cell temperature was kept at 25 °C, Table S1: Average position and atm % of the samples from 3 high resolution Pt 4f XPS spots, Table S2: Average position and atm % of the samples from 3 high resolution Ni 3p XPS spots, Table S3: Ex-situ RDE measurement quantitative data.
