# Supplementary material for: Annealing Behaviour of Pt and PtNi Nanowires for Proton Exchange Membrane Fuel Cells
Source: Materials (Basel). 2018 Aug 19;11(8):1473. doi: 10.3390/ma11081473 (PMC6120042; doi:10.3390/ma11081473)
Supplement: Supplementary file 1 [file materials-11-01473-s001.pdf]

Supplementary Information for:

Annealing Behaviour of Pt and PtNi Nanowires for Proton Exchange Membrane Fuel Cells

Peter Mardle and Shangfeng Du*

School of Chemical Engineering, University of Birmingham, Edgbaston, Birmingham,

B15 2TT, UK

* Corresponding author, Tel: +44 121 4158696

E-mail address: s.du@bham.ac.uk (S. Du)

1. MEA Testing protocol

1.1 Hydration and cell break-in

All MEAs were initially heated to 80 °C held at 0.6 V for at least 6 hrs and up until a constant current was observed under a cathode/anode gas supply of air/H_2 respectively. The cathode/anode relative humidity (RH), stoichiometric coefficient and absolute pressure was 100 %/100 %, 1.5/1.3 and 1.3 bar/1.5 bar respectively. The required conditions for the polarisation curve were then set as detailed in the experimental section of the main report before running the following break in procedure: The current was held at 11.2 A for 5 minutes before holding the potential at 0.8 V for 5 mins, 0.4 V for 5 mins, 0.8 V for 5 mins, 0.6 V for 5 mins, 0.8 V for 5 mins and then 11.2 A for 35 mins, ensuring stability of the recorded voltage.

1.2 Polarisation curve acquisition

Polarisation curves were obtained using the procedure detailed in Appendix E of the harmonised protocols [1].

References

[1] Tsotridis G, Pilenga A, Marco G De, Malkow T. EU Harmonised Test Protocols for PEMFC MEA Testing in Single Cell Configuration for Automotive Applications; JRC Science for Policy report. 2015. doi:10.2790/54653.

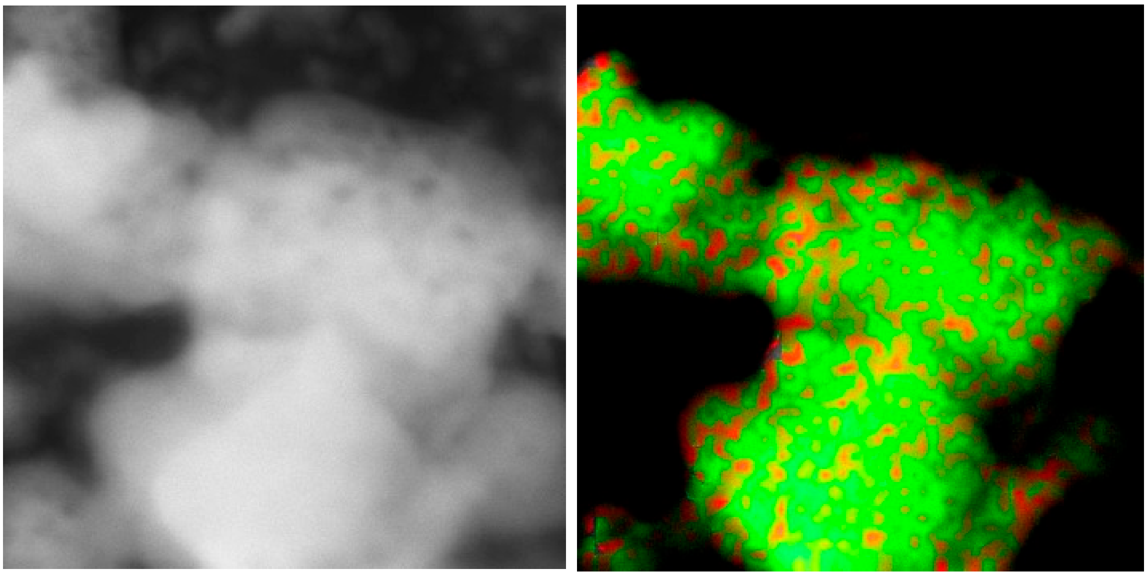

Figure S1: STEM mode image and EDX element map of PtNi NWs/C annealed at 350 ^oC. The EDX map is of Pt (green) and Ni (red).

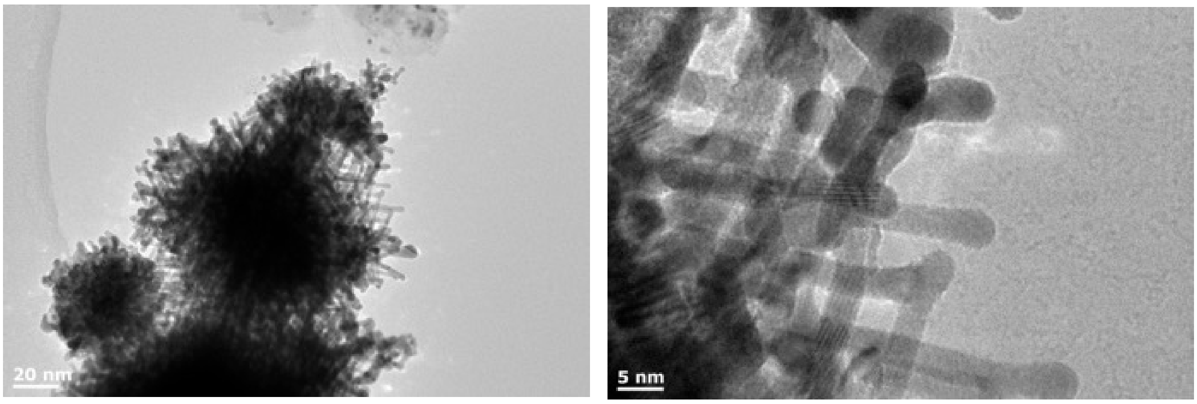

Figure S2: TEM of PtNi NWs/C (150 ^oC, 72 hrs).

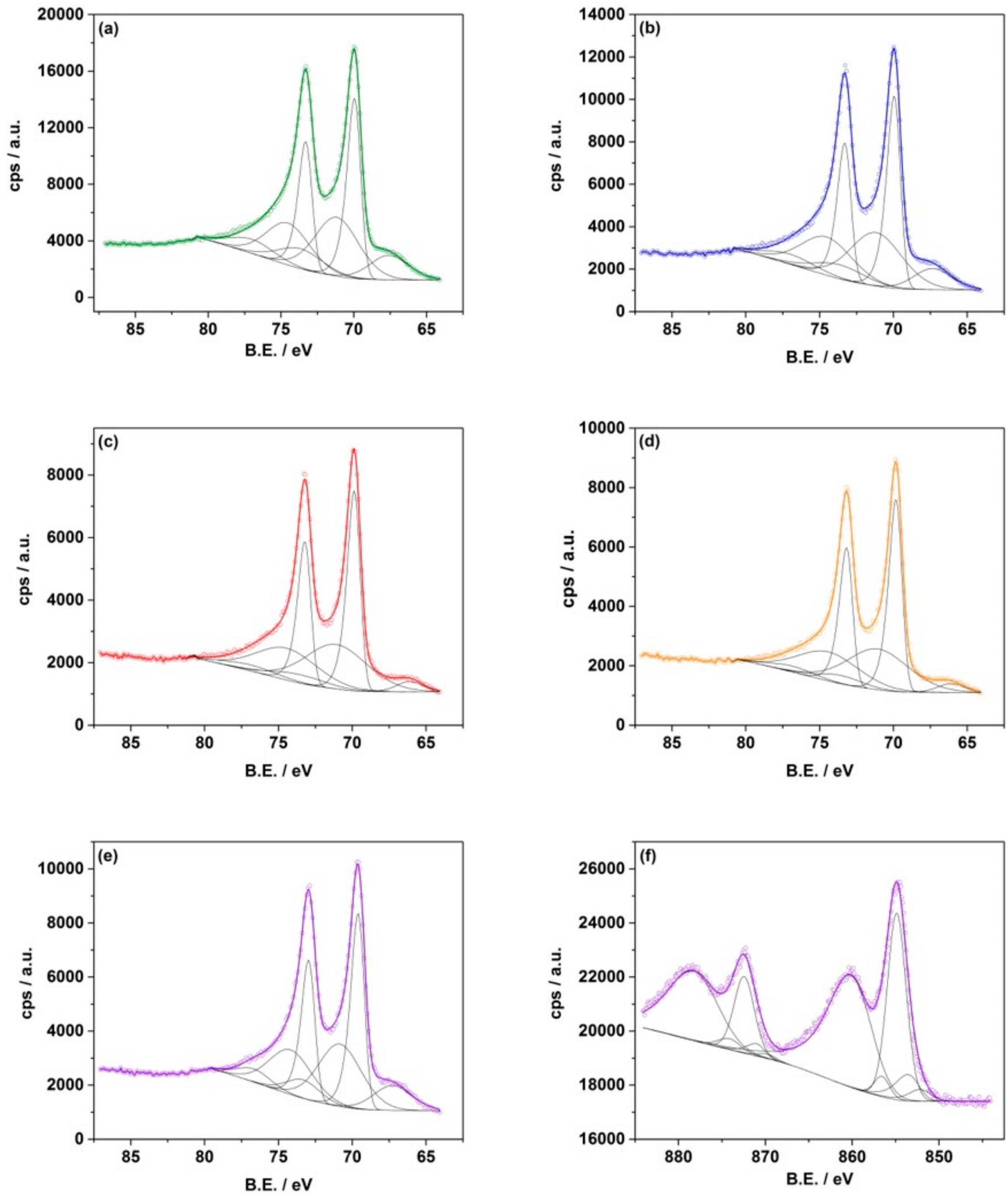

Figure S3: XPS patterns of the Pt 4f regions of **(a)** PtNi NWs/C, **(b)** PtNi NWs/C (150 ^{o}C), **(c)** PtNi NWs/C (250 ^{o}C), **(d)** PtNi NWs/C (350 ^{o}C), **(e)** PtNi NWs/C (150 ^{o}C, 72 hrs) and **(f)** the Ni 3p region of PtNi NWs/C (150 ^{o}C, 72 hrs).

Table S1: Average position and atm % of the samples from 3 high resolution Pt 4f XPS spots.

Sample		Average Position / eV (and atm %)					
	Pt(0) 7/2	Pt(0) 5/2	Pt(II) 7/2	Pt(II) 5/2	Pt(IV) 7/2	Pt(IV) 5/2	Ni 3p
**Pt NWs/C**	69.93 (28.06)	73.28 (20.02)	71.33 (20.32)	74.68 (14.50)	73.83 (9.98)	77.18 (7.12)	- (-)
**PtNi NWs/C**	69.73 (16.24)	73.08 (11.59)	71.13 (13.66)	74.48 (9.75)	73.63 (5.88)	76.98 (4.20)	67.60 (38.67)
**PtNi NWs/C (150 °C)**	69.73 (17.28)	73.08 (12.33)	71.13 (16.55)	74.48 (11.81)	73.63 (4.57)	76.98 (3.26)	67.34 (34.19)
**PtNi NW/C (150 °C, 72 hrs)**	69.42 (16.94)	72.77 (12.09)	70.82 (16.93)	74.17 (12.08)	73.32 (3.65)	76.67 (2.60)	67.06 (35.71)
**PtNi NW/C (250 °C)**	69.58 (23.49)	72.93 (16.76)	70.98 (22.75)	74.33 (16.24)	73.48 (3.08)	76.83 (2.20)	66.08 (15.50)
**PtNi NWs/C (350 °C)**	69.60 (25.29)	72.95 (18.05)	71.00 (22.55)	74.35 (16.09)	73.50 (3.29)	76.85 (2.35)	66.10 (12.38)

Table S2: Average position and atm % of the samples from 3 high resolution Ni 3p XPS spots.

Sample	Average Position / eV (and atm %)									
	Ni 3/2	NiO 3/2	$Ni(OH)_2$ 3/2	NiOOH 3/2	Ni Sat' 3/2	Ni 1/2	NiO 1/2	$Ni(OH)_2$ 1/2	NiOOH 1/2	Ni Sat' 1/2
**Pt(NW)/C**	- (-)	- (-)	- (-)	- (-)	- (-)	- (-)	- (-)	- (-)	- (-)	- (-)
**Pt NWs/C**	852.21 (2.05)	853.31 (1.82)	855.11 (20.59)	856.81 (2.68)	859.55 (40.85)	870.18 (0.33)	871.28 (2.59)	873.08 (8.09)	874.78 (1.37)	877.81 (19.63)
**PtNi NWs/C (150 °C)**	852.14 (2.93)	853.24 (3.27)	855.04 (18.91)	856.74 (3.53)	859.41 (36.75)	869.97 (0.66)	871.07 (1.73)	872.87 (10.40)	874.57 (1.44)	878.10 (20.37)
**PtNi NWs/C (150 °C, 72 hrs)**	851.97 (1.65)	853.07 (5.63)	854.87 (20.62)	856.57 (1.82)	859.25 (33.01)	869.88 (0.69)	870.98 (3.63)	872.78 (9.25)	874.48 (1.50)	877.75 (22.19)
**PtNi NWs/C (250 °C)**	851.71 (8.07)	852.81 (5.99)	854.61 (10.85)	856.31 (1.02)	858.60 (33.09)	869.45 (5.52)	870.55 (3.60)	872.35 (4.00)	874.05 (0.87)	877.15 (27.00)
**PtNi NWs/C (350 °C)**	851.62 (6.71)	852.72 (5.10)	854.52 (10.13)	856.22 (1.35)	858.63 (30.14)	869.61 (6.80)	870.71 (2.37)	872.51 (4.59)	874.21 (0.99)	877.33 (31.81)

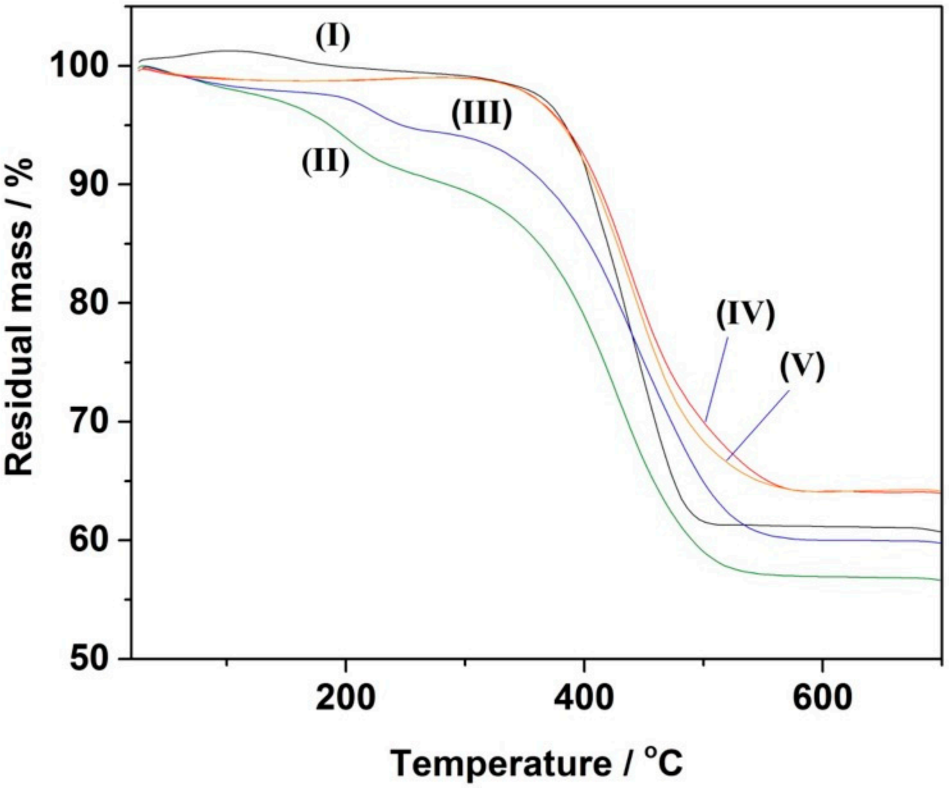

Figure S4: FFT 5 point smoothed TGA of (I) Pt NWs/C, (II) PtNi NWs/C, (III) PtNi NWs/C (150 °C), (IV) PtNi NWs/C (250 ^{O}) and (V) PtNi NWs/C (350 °C).

Table S3: Ex-situ RDE measurement quantitative data.

Sample	$i_{k\ 0.9V}$ / mA cm^{-2}	ECSA / $m^2\ g_{Pt}^{-1}$	Specific activity / μA cm_{Pt}^{-2}	Mass activity / A mg_{Pt}^{-1}
**Pt/C (TKK)**	14.62	85.7	292	0.250
**Pt NWs/C**	5.37	13.1	536	0.070
**Pt NWs/C (150 °C)**	5.01	8.1	808	0.066
**Pt NWs/C (250 °C)**	3.27	7.6	561	0.043
**Pt NWs/C (350 °C)**	4.50	6.1	968	0.059
**PtNi NWs/C**	4.45	8.0	769	0.062
**PtNi NWs/C (150 °C)**	9.00	11.1	1123	0.125
**PtNi NWs/C (150 °C, 72 hrs)**	5.96	9.3	889	0.083
**PtNi NWs/C (250 °C)**	2.99	4.8	855	0.041
**PtNi NWs/C (350 °C)**	4.87	4.2	1606	0.067

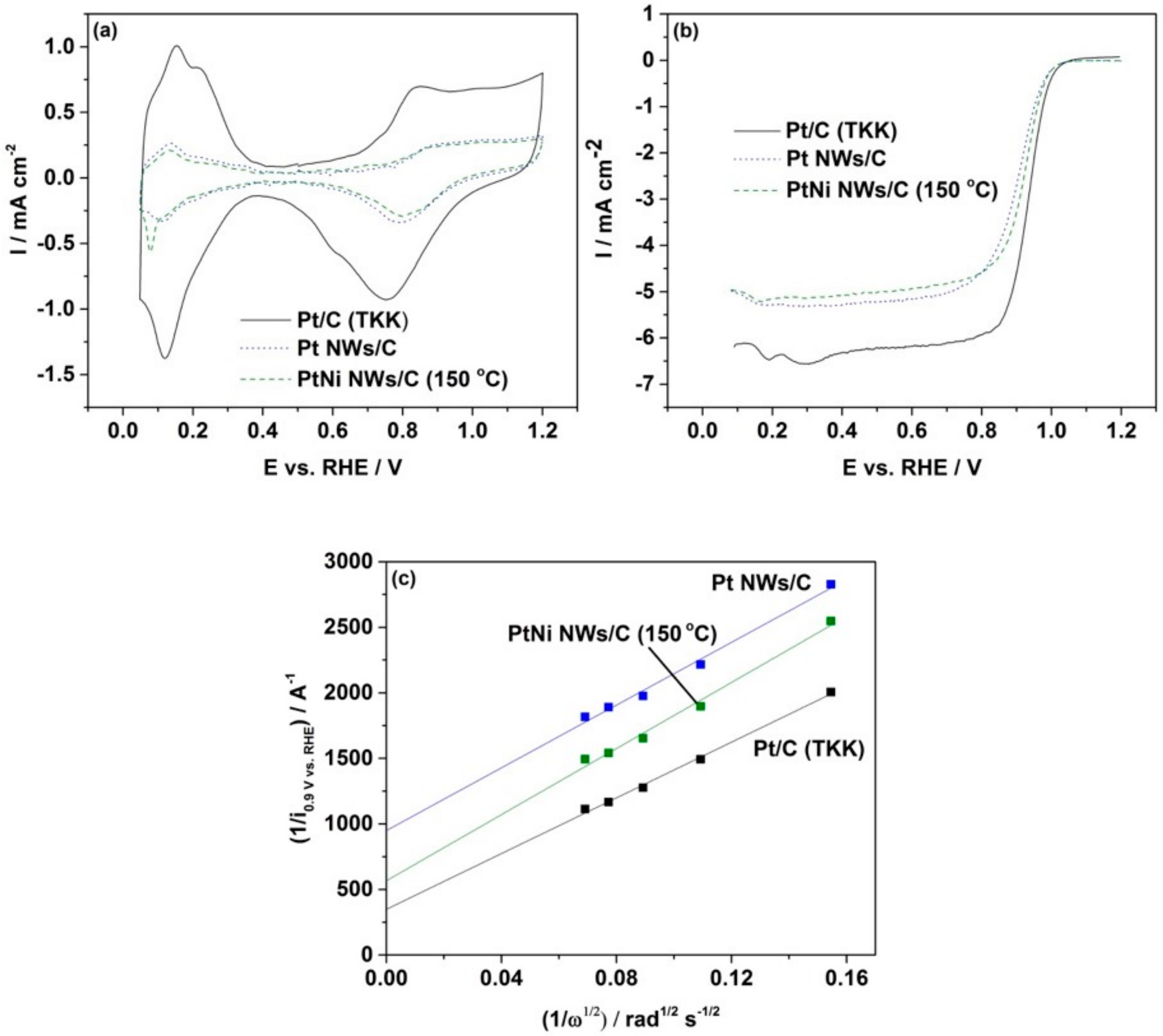

Figure S5: (a) CVs of 25 μg catalyst on a 0.196 cm^2 GCE in N_2 saturated 0.1 M $HClO_{4(aq)}$ electrolyte in the potential range 0.05-1.2 V. **(b)** LSVs at 1600 rpm in O_2 saturated electrolyte from 0.05-1.2 V vs. RHE with a sweep rate of 20 mV s^{-1}. **(c)** Koutecky-Levich plots. The cell temperature was kept at 25 ^{o}C.
